# Removal of Cu(II) from Aqueous Medium Using Chitosan/*n*SiO_2_ Nanocomposite: Fixed-Bed Adsorption Study and Modeling

**DOI:** 10.3390/polym18212791

**Published:** 2026-11-08

**Authors:** Samanwita Bhattacharya, Nirjhar Bar, Baisali Rajbansi, Sudip Kumar Das

**Affiliations:** 1 Department of Chemical Engineering, University of Calcutta, 92, A. P. C. Road, Kolkata 700009, Indianirjhar@hotmail.com (N.B.); brbchemengg@caluniv.ac.in (B.R.); 2 St. James’ School, 165, A. J. C. Bose Road, Kolkata 700014, India

**Keywords:** chitosan, nanocomposite, breakthrough curve, Thomas model, genetic algorithm

## Abstract

The study aims to use Chitosan/*n*SiO_2_ Nanocomposite (CSNC) as an adsorbent to eliminate toxic Cu(II) ions from its aqueous solution using a fixed-bed continuous column system. *n*SiO_2_ was synthesized from rice husk ash. The adsorption investigations were performed at optimum pH 6.5 under different operating conditions, such as bed height (3–7 cm), influent flow rates (30–50 mL min^−1^), and influent Cu(II) concentration (10–30 mg L^−1^). The removal of Cu(II) was increased with the column’s bed depth but reduced with flow rate and adsorbate concentration. Experimental observations were employed to study seven kinetic models. The Thomas model was confirmed to be the best-fit kinetic model corresponding to 3 cm bed height, a column diameter of 1.5 cm, 30 mL min^−1^ flow rate, and an initial metal concentration of 30 mg L^−1^. A regeneration study and scale-up design were also reported. MLR (R^2^ > 0.98) and GA techniques (R^2^ = 1) were also applied to forecast the elimination of Cu(II). The performance of the prepared nanocomposite adsorbent was evaluated on a laboratory scale for removing Cu(II) from synthetic wastewater. This study integrates sustainability and circular economy principles in the application of adsorbents by focusing on their synthesis, utilization in fixed-bed continuous column systems, regeneration, and safe disposal.

## 1. Introduction

Over the years, heavy metals, dyes, organics, and pharmaceuticals in wastewater have been the foremost contributing factors to the declining quality of the environment [1]. Being one of the priorities technophilic heavy metals, copper is a significant threat to the ecological environment. Apart from various industrial and metallurgical processes, corrosion of household plumbing systems is another significant contributing factor towards copper contamination. Through various investigations with symptoms and diagnoses, a clear image of the severe adverse effects of copper toxicity on human health has come into view. Chronic Cu (II) exposure may be linked to Wilson’s disease, described by hepatic cirrhosis, severe damage to the kidney and brain, and accumulation of Cu(II) in the cornea. Occupational exposure often causes metal fever. Long-term exposure may result in nasal, mucous, and ophthalmic irritations, headaches, stomachaches, dizziness, vomiting, and diarrhea [2]. In drinking water, the maximum contaminant level for Cu(II) is 1.3 mg L^−1^ [3]. Hence, it is essential to eliminate these toxicants from wastewater before releasing them into water streams through a properly investigated treatment process using newer adsorbents, which must be sustainable in terms of the adsorption performance, cost, regeneration, and safe disposal after use [4].

Amongst various conventional purification techniques, viz. micro electrolysis [5], nanofiltration [6], and coagulation–ultrafiltration [7], adsorption has proven to be the most extensively employed approach due to its high competence, convenient operation, simple design, and cost-effectiveness. Recent studies focus on the development of adsorbents of biopolymer origin. The use of chitosan (CS) as a biosorbent has achieved prominent attention due to its fascinating physicochemical characteristics, viz. non-toxicity, bio-compatibility, bio-degradability, bio-functionality, outstanding chelating properties, and excellent selectivity to various pollutants [8,9]. With the relevance of nanotechnology in wastewater treatment, several hybrid nano-sized adsorbents based on CS modified with polymers have been explored for metal removal: zeolite–CS nanocomposite [10]; saw dust/CS nanocomposite [11]; Polymethacrylic acid and nano-sized halloysite nanotube (which was easily crosslinked with CS) [12]; hydrogel nanocomposite based on CS-*g*-acrylic acid (chitosan-grafted poly acrylic acid) and modified nano silica [13]; magnetic nanocomposite of aminopropyl triethoxysilane coated with carboxymethyl cellulose crosslinked with CS nanoparticles [14]; DTPA-modified CS/polyethylene oxide nanofiber [15]; polyacrylic acid-grafted magnetic CS nanocomposite [16]; and magnetic CS nanocomposites modified with graphene oxide and polyethyleneimine [17] as well as metals and metal oxides, like CS–nano-TiO_2_ [18], CS–zero-valent iron nanocomposite [19], triethylenetetramine-modified hollow Fe_3_O_4_/SiO_2_/CS magnetic nanocomposite [20], MnO_2_/CS nanocomposite [21], CS–silver nanocomposite [22], SnO_2_-crosslinked-chitosan nanocomposite [23], and ZnO@CS core–shell nanocomposite [24], etc. But, unfortunately all of the above materials are not easy to synthesize and regenerate. In addition, they are not cost-effective for third world countries. Moreover, the application of continuous column adsorption systems using these materials is not well reported. Hence, advancements in techniques and the development of new adsorbent materials are of interest to various researchers.

Multiple linear regression (MLR) is an important statistical technique for establishing quantitative relationships between independent variables and one or more dependent variables. The Genetic Algorithm (GA), inspired by the principles of natural genetics and biological evolution, is an evolutionary optimization technique widely employed to solve complex optimization problems, including multi-objective optimization [18,25,26]. In recent years, hybrid approaches integrating Genetic Algorithms with artificial neural networks have gained considerable attention as effective tools for model optimization and predictive performance enhancement [27].

Considering the good performance of the adsorbent CS/nanoSiO_2_ nanocomposite (CSNC) in the batch study [25], it has been utilized in fixed-bed down-flow continuous column systems for Cu(II) adsorption under variable experimental conditions. Although the fixed-bed continuous column adsorption method is superior to a batch process under continuous flow conditions when treating large volumes of industrial effluent [28,29], only a few investigations have reported on fixed-bed adsorptive removal of Cu(II) by chitosan-based nanocomposites [30]. Therefore, the present study aims to address this research gap by utilizing rice husk ash, an indigenous waste material, as a precursor for the synthesis of nano silica [31]. In addition, the regeneration of spent adsorbents and the scale-up design of the adsorption process are discussed in detail. Multiple linear regression (MLR) and hybrid modelling techniques are also employed to predict Cu(II) removal efficiency.

## 2. Materials and Methods

### 2.1. Chemicals Used

All chemicals used in this study were of analytical grade. Low-molecular-weight, extra-pure chitosan powder (degree of deacetylation ≥ 90%, CAS 9012-76-4) was procured from SRL Pvt. Ltd., West Bengal, India. The other chemicals, including acetic acid, 25% glutaraldehyde solution, ethanol, copper sulfate pentahydrate (CuSO_4_·5H_2_O), sodium hydroxide pellets, and hydrochloric acid were purchased from E. Merck India Ltd., Mumbai, India Doubly distilled water was used throughout the experimental procedures. Silica nanoparticles were synthesized using rice husk ash as the precursor, following the previously reported method [31].

### 2.2. Methods

#### 2.2.1. Adsorbent Preparation

In our previous study, CS/*n*SiO_2_ nanocomposite adsorbents with different chitosan to nano silica weight ratios were synthesized and evaluated. Among the prepared composite, CS:*n*SiO_2_, with a weight ratio of 2:1 (CSNC2-1), exhibited the best performance for Cu(II) removal in batch adsorption experiments [25]. Therefore, CSNC2-1 was selected as the adsorbent for the subsequent fixed-bed column studies.

#### 2.2.2. Instruments Used for the Characterization of Adsorbent

SEM, FTIR, BET, XRD, TGA, and point of zero charge (pH_PZC_) were measured [25]. The pH was measured using a WTW pH meter (Multi 340i/SET of Germany).

#### 2.2.3. Preparation of Cu(II) Solution

A stock solution of Cu(II) with a concentration of 1000 mg L^−1^ was prepared by dissolving 3.929 g of CuSO_4_·5H_2_O in 1 L of distilled water. The stock solution was subsequently used to obtain experimental solutions of the desired Cu(II) concentrations.

#### 2.2.4. Column Adsorption Experiments

**Figure 1:**
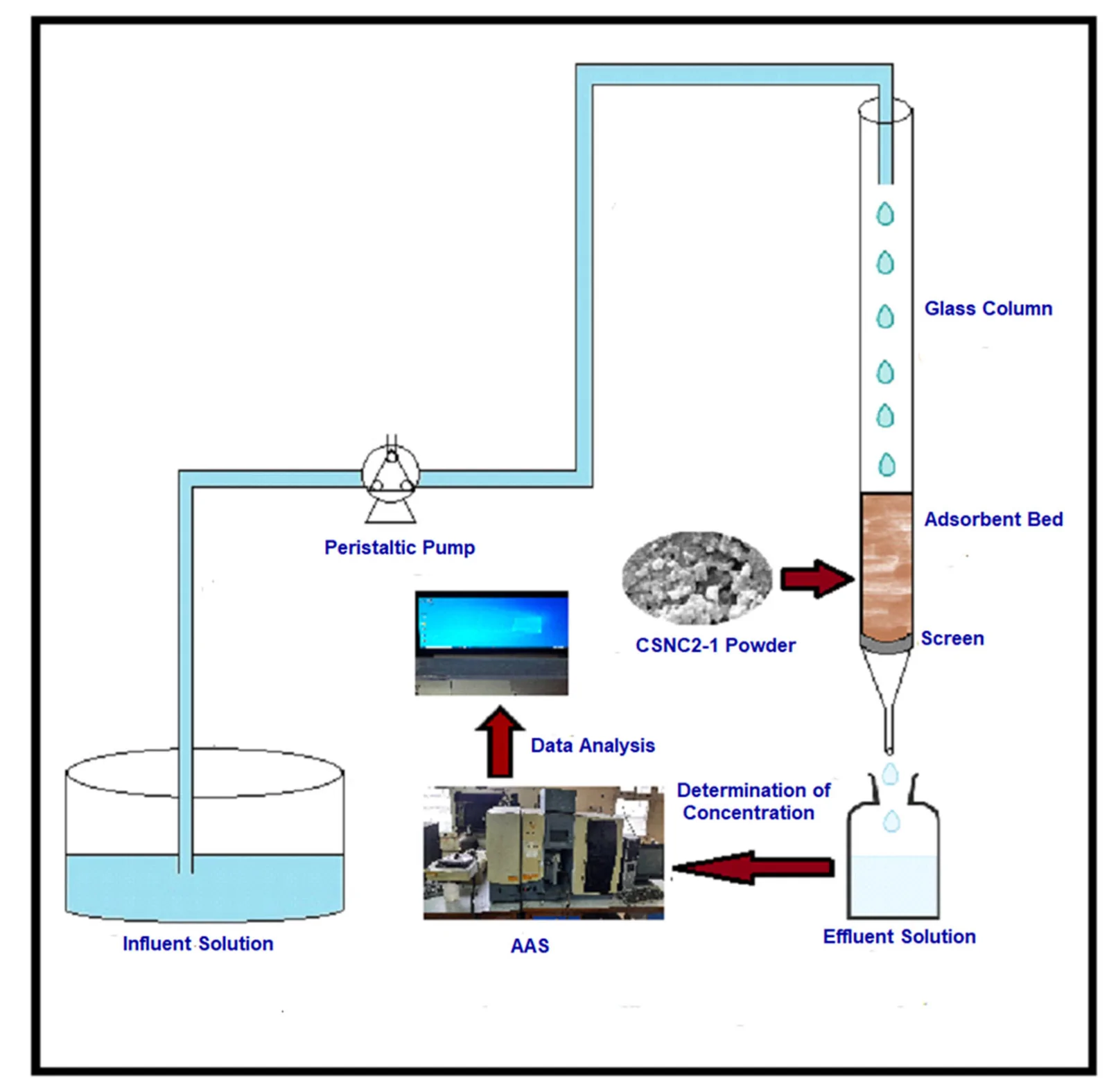
Schematic representation of the experimental set-up.

Cu(II) adsorption from aqueous solutions was investigated using a laboratory-scale, continuous down-flow fixed-bed column system under different experimental conditions. The experimental setup consisted of three glass columns, each measuring 33 cm in length and 1.5 cm in internal diameter. A stainless-steel sieve plate was placed at the bottom of each column to support the adsorbent bed, with a thin layer of glass wool positioned above it. The columns were packed with the required quantities of adsorbent to achieve bed depths of 3, 5, and 7 cm. The influent Cu(II) solution was continuously introduced into the columns in a downward direction using a peristaltic pump (Model 7535-04, Cole-Parmer, Vernon Hills, IL, USA). The experiments were conducted at pH 6.5, as optimized in the batch adsorption study [25], and at room temperature. Effluent samples were collected at predetermined time intervals and analyzed for residual Cu(II) concentrations using an atomic absorption spectrophotometer (AAS; AA 240 VARIAN, Sydney, Australia), following the standard analytical procedure [32]. A schematic diagram of the fixed-bed column adsorption system is presented in Figure 1. The effects of key operating parameters, namely bed depth, initial Cu(II) concentration, and flow rate, on the adsorption performance were systematically investigated while maintaining the optimum pH identified in the batch study and a constant room temperature.

Equations (1)–(3) were used to calculate the percentage removal efficiency [27,33].

(1) $$\%\;\mathrm{removal}=\frac{q_{\mathrm{total}}}{m_{\mathrm{total}}}\times 100$$

where

(2) $$q_{\mathrm{total}}=QC_{0}\int_{0}^{t_{\mathrm{total}}}\left(1-\frac{C_{t}}{C_{0}}\right)\mathrm{dt}$$

and

*m_total_* = *QC*_0_*t_total_* (3)

Equation (4) was used to calculate the experimental uptake capacity *q_e_*_(exp)_ [33,34].

(4) $$q_{e(\exp)}=\frac{q_{\mathrm{total}}}{m_{d}}$$

## 3. Results and Discussion

### 3.1. Characterization of Adsorbent

The results of SEM analysis, FTIR studies, BET analysis, XRD studies, TGA, and PZC analysis were reported in detail in our previous work [25]. The porosity of the bed varies from 0.28 to 0.31 for different bed heights and also with the same bed height for different operating conditions. The density of the adsorbent is 1.67 g/cc, the surface area is 166 m^2^/g from the BET study [25], and the bulk density is 0.738 g/cc.

### 3.2. Influences of Operating Parameters on Fixed-Bed Adsorption

#### 3.2.1. Effects of Bed Depth

**Figure 2:**
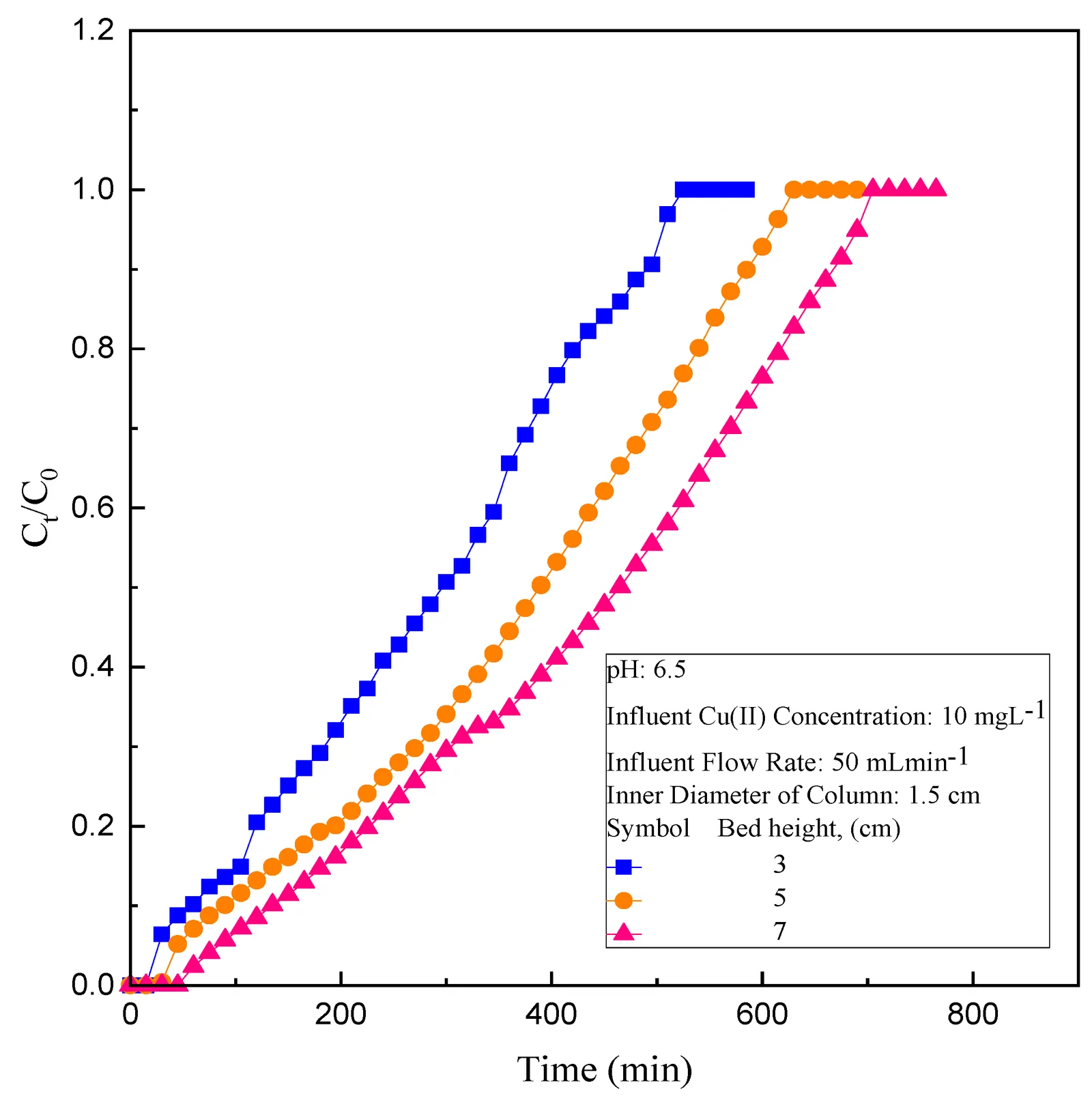
Effect of variation in bed depth.

The effect of bed depth on Cu(II) adsorption was investigated at bed depth heights of 3, 5, and 7 cm while maintaining a constant influent flow rate of 50 mL min^−1^ and an initial Cu(II) concentration of 10 mg L^−1^. The corresponding breakthrough curves are shown in Figure 2. The results demonstrated that increasing bed depth improves Cu(II) removal and prolonged both the breakthrough time (C_t_/C_0_ = 0.05) [35] and the exhaustion time (C_t_/C_0_ = 0.95) [35]. The breakthrough and exhaustion times increased from 26.6 to 81.2 min and from 505.7 to 692.8 min, respectively, with increasing bed height. This improvement can be attributed to the greater availability of adsorption sites and the increased contact with Cu(II) ions and the adsorbent particles. Furthermore, the longer residence time is associated with a deeper bed, which facilitates enhanced adsorption [27,36].

However, increasing the bed height also resulted in a broader mass transfer zone, which may be attributed to increased mass transfer limitations and prolonged progression of adsorption kinetics. These limitations were associated with repulsive interaction between Cu(II) ions adsorbed onto the CSNC2-1 surface and those remaining in the aqueous phase [37,38]. In contrast, diffusion becomes more dominant than axial dispersion at greater bed heights [37,39], indicating a change in the relative contributions of these transport mechanisms to the overall adsorption process.

#### 3.2.2. Effects of Influent Flow Rate

**Figure 3:**
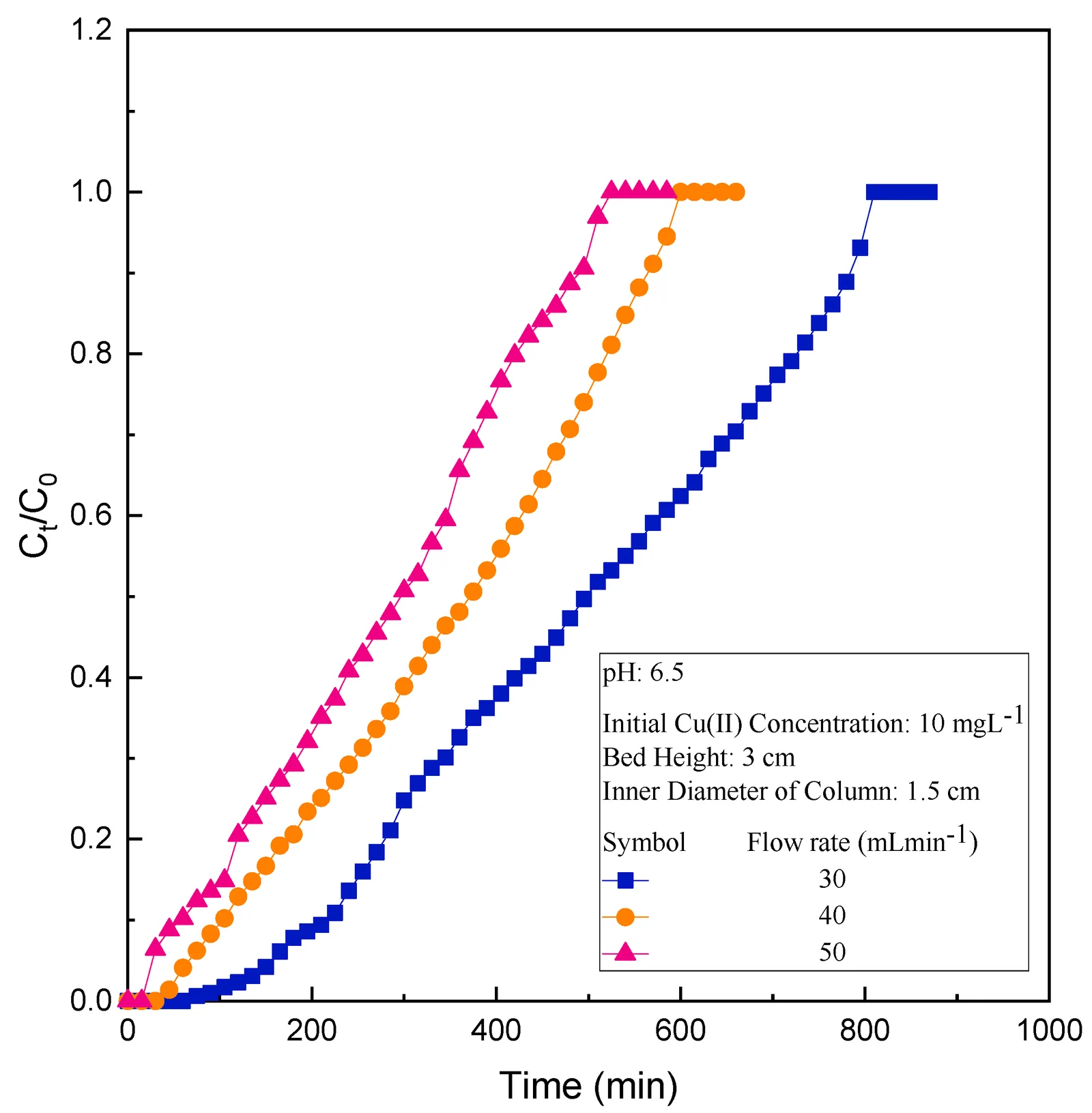
Effect of influent flow rate.

The adsorption experiments were conducted at different influent flow rates from 30 to 50 mL mim^−1^, while maintaining a constant bed height of 3 cm and an influent Cu(II) concentration of 10 mg L^−1^. The corresponding breakthrough curves are presented in Figure 3. As the influent flow rate increased, the Cu(II) removal efficiency decreased, and the breakthrough and exhaustion times decreased from 158.2 to 25.2 min and from 800.7 to 506.1 min, respectively. This behavior can be attributed to the reduced contact time between the Cu(II)-containing solution and the adsorbent at higher flow rates, which limits the effectiveness of the adsorbent. Furthermore, the increased flow rate reduces the time available for Cu(II) ions to migrate from the bulk aqueous phase to the adsorbent surface, thereby influencing external film mass transfer. At lower flow rates, the longer residence time facilitates intraparticle diffusion, allowing Cu(II) ions to penetrate further into the adsorbent pores [37,38,40].

#### 3.2.3. Effects of Influent Cu(II) Concentration

**Figure 4:**
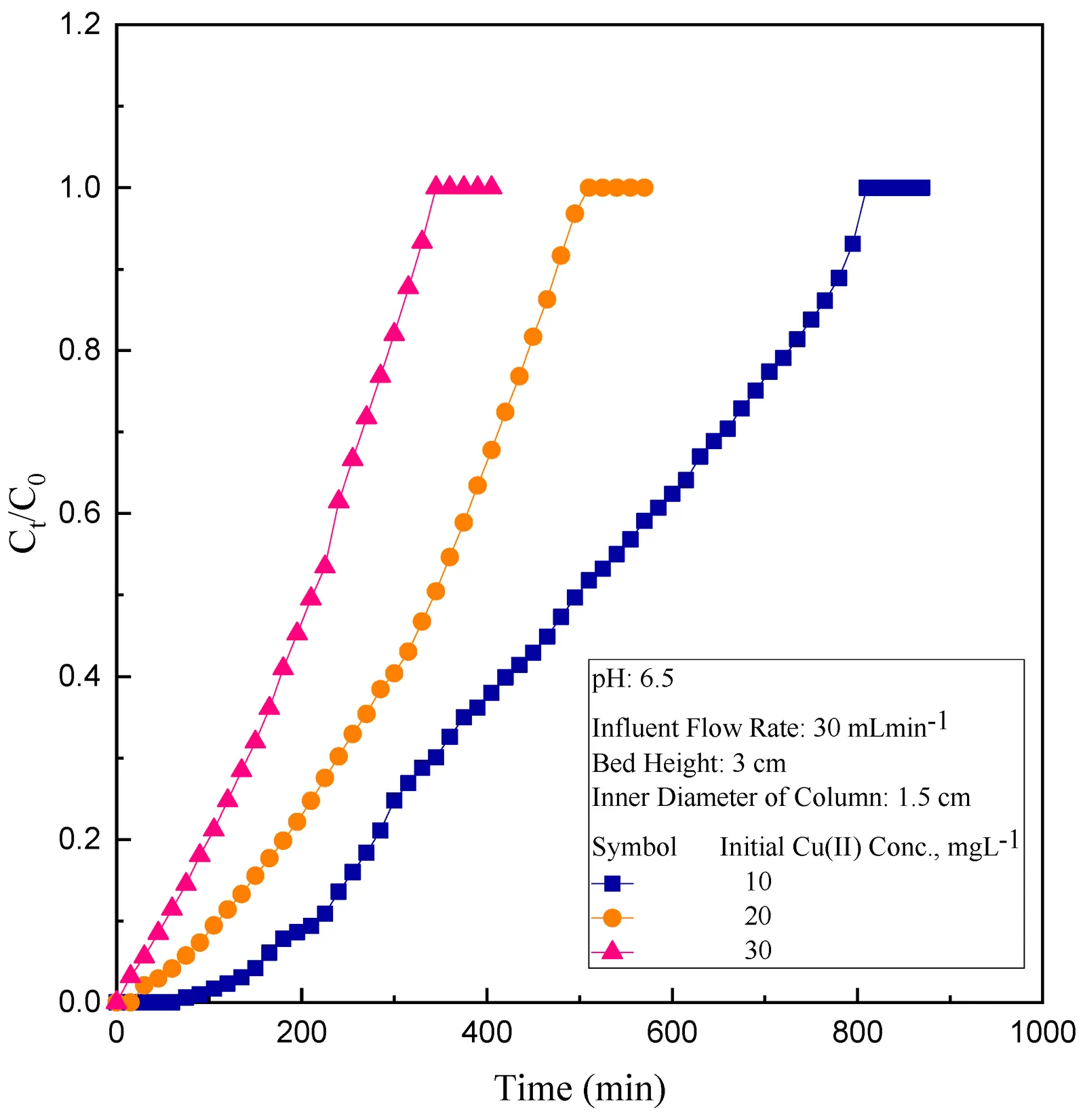
Effect of initial Cu(II) concentration.

The adsorption investigations were carried out with several influent Cu(II) concentrations (10–30 mg L^−1^), with a constant bed height of 5 cm and a constant flow rate of 30 mL min^−1^. The breakthrough curves are shown in Figure 4. As the Cu(II) concentration increased, the breakthrough and exhaustion periods were reduced from 158.9 min to 23.1 min and 799.4 min to 337.3 min, respectively, due to a faster diffusional mass transfer and an increased rate of binding site saturation at higher Cu(II) concentrations [27,38,40]. Prolonged transport was observed due to a decrease in the diffusion coefficient or the mass transfer coefficient at a low concentration gradient [41].

### 3.3. Kinetic Study

Seven kinetic models were fitted to the experimental data to evaluate the adsorption kinetics. The estimated kinetic parameters, correlation coefficients (R^2^), sum of squared errors (SSE), standard deviation (SD) values, and corresponding kinetic equations, along with their interpretations, are summarized in Table 1.

Considering the R^2^ and lower SD values, the experimental results fit well in the Yan and Modified dose–response models. However, the values of maximum adsorption capacities obtained from these two models differ from the experimentally observed values of adsorption capacities. Therefore, the fitting of these two models could be more satisfactory due to the anomalous trend of model parameters.

This drawback of the prediction of maximum adsorption capacities can be overcome using the Thomas model, which can be further used for scale-up design.

### 3.4. Scale-Up Design

Evaluating the scale-up design is essential for translating laboratory-scale findings into practical applications. In the present study, the scale-up design of the fixed-bed column system was developed based on the best-fitting kinetic model.

The Thomas model exhibited the best fit to the experimental data under the following operating conditions: a bed height of 3 cm, an inner column diameter of 1.5 cm, an influent flow rate of 30 mL min^−1^, and an influent Cu(II) concentration of 30 mg L^−1^.

Equations (5)–(8) [27,40] were essential to estimate the parameters given in Table 2 for scale-up design:

(5) $$Q_{d}=\frac{\pi D^{2}U_{0}}{4}$$

where

(6) $$D=\sqrt{\frac{4W}{\pi t_{W}U_{0}}}$$

(7) $$M_{\mathrm{ad}}=\frac{Q_{d}C_{w}}{q_{\mathrm{ad}}}\int_{0}^{t_{R}}\left(1-\frac{C_{t}}{C_{w}}\right)\mathrm{dt}$$

(8) $$H=H_{c}+\frac{4M_{\mathrm{ad}}}{\pi D^{2}\rho_{\mathrm{ad}}}$$

These data should be optimized for industrial purposes by modifying the column parameters according to the standard design for pumps and vessels.

### 3.5. Safe Disposal of Used Adsorbent

The Cu(II)-loaded adsorbent was not disposed of in the open environment due to the risk of Cu(II) leaching. No detectable Cu(II) leaching was observed after incinerating the spent adsorbent at temperatures above 600 °C and subsequently mixing the resulting ash with water at a 1:5 ratio for 24 h [27]. Therefore, the ash could be safely utilized in road repair, land-filling, and brick manufacturing.

### 3.6. Regeneration Study

**Figure 5:**
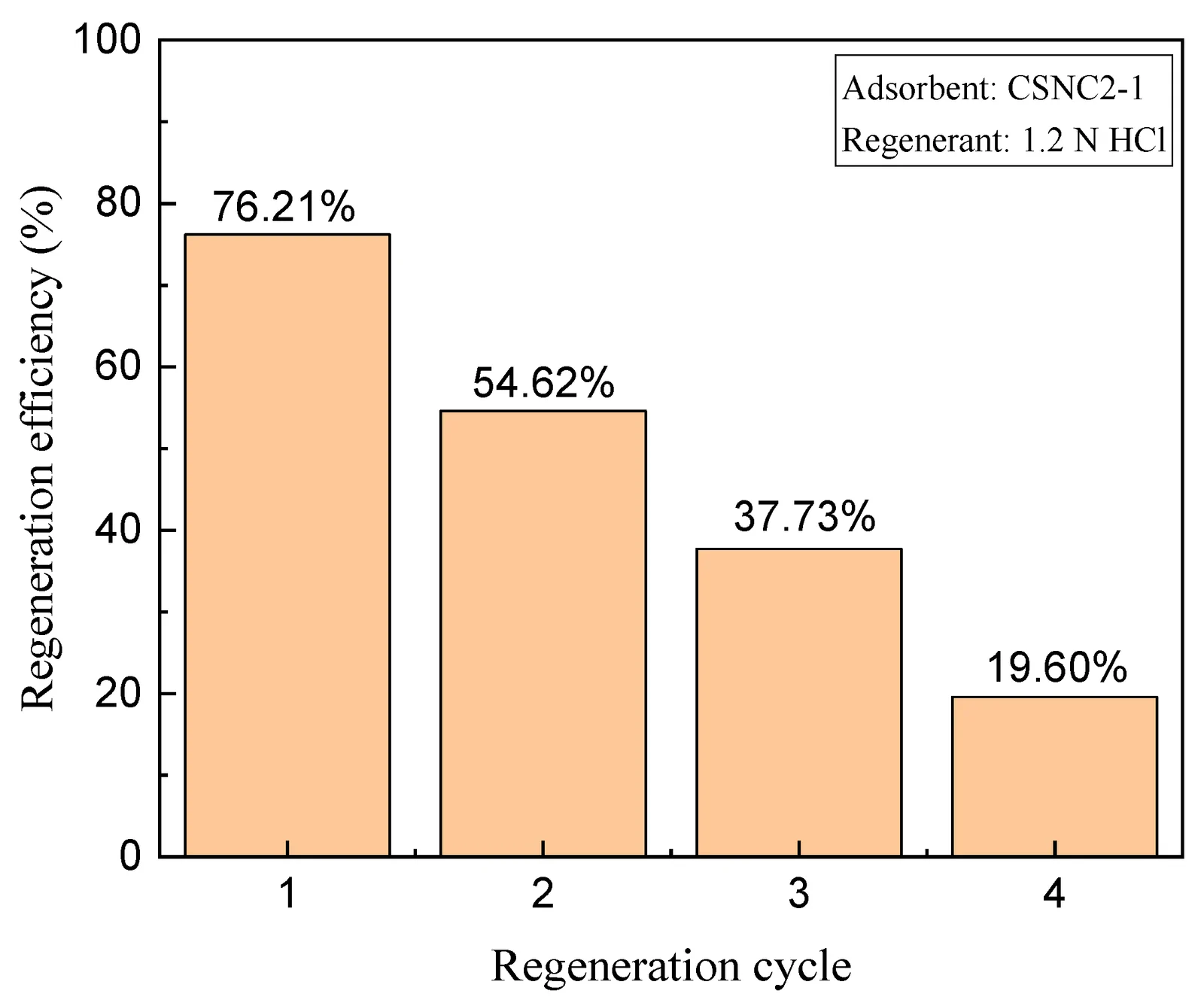
Regeneration efficiency of CSNC2-1 after each regeneration cycle.

Figure 5 shows that the effective regenerant for Cu(II) adsorption was 1.2 N HCl using the 3 cm bed depth with an initial Cu(II) concentration of 10 mg L^−1^ and an influent flow rate of 30 mL min^−1^. The regeneration efficiency (*ε*) and capacity, *q_R_* were calculated from the following equations:

(9) $$\varepsilon =\frac{q_{R}}{q_{\mathrm{total}}}\times 100\%$$

(10) $$q_{R}=Q\int_{0}^{t_{\mathrm{RS}}}\left(C_{0}-C_{t}\right)\mathrm{dt}$$

Regeneration efficiency was calculated to be 76.21% to 19.60% from the first to fourth cycles, respectively.

### 3.7. Regression Analysis

For the analysis concerning the rate expression associated with MLR, the independent variables are flow rate (*Q*), bed height (*Z*), time (*t*), influent concentration (*C_O_*), and residual concentration (*C_t_*), and the dependent variable is elimination (%) of Cu(II) ($PR_{\mathrm{Col}-\mathrm{Si}O_{2}-\mathrm{Cu}(\mathrm{II})}$). The data accumulated from the experimentation process to predict $PR_{\mathrm{Col}-\mathrm{Si}O_{2}-\mathrm{Cu}(\mathrm{II})}$ through MLR is detailed in Table 3.

The first-degree polynomial equation [Equation (11)] is expressed to eliminate Cu(II) competence of the adsorbent CSNC2-1.

(11) $$\begin{array}{ll} PR_{\mathrm{Col}-{\mathrm{SiO}}_{2}-\mathrm{Cu}(\mathrm{II})} & =1.17755-0.203763\times Q+0.162581\times Z\\ & -0.880945\times t-0.073358\times C_{O}-0.727398\times C_{t} \end{array}$$

The variance, mean squared error, and correlation coefficient obtained from Equation (11) are 0.03452, 0.001192, and 0.989776, respectively, having a *t*-value of 1.9686 for 276 degrees of freedom within 95% confidence.

**Figure 6:**
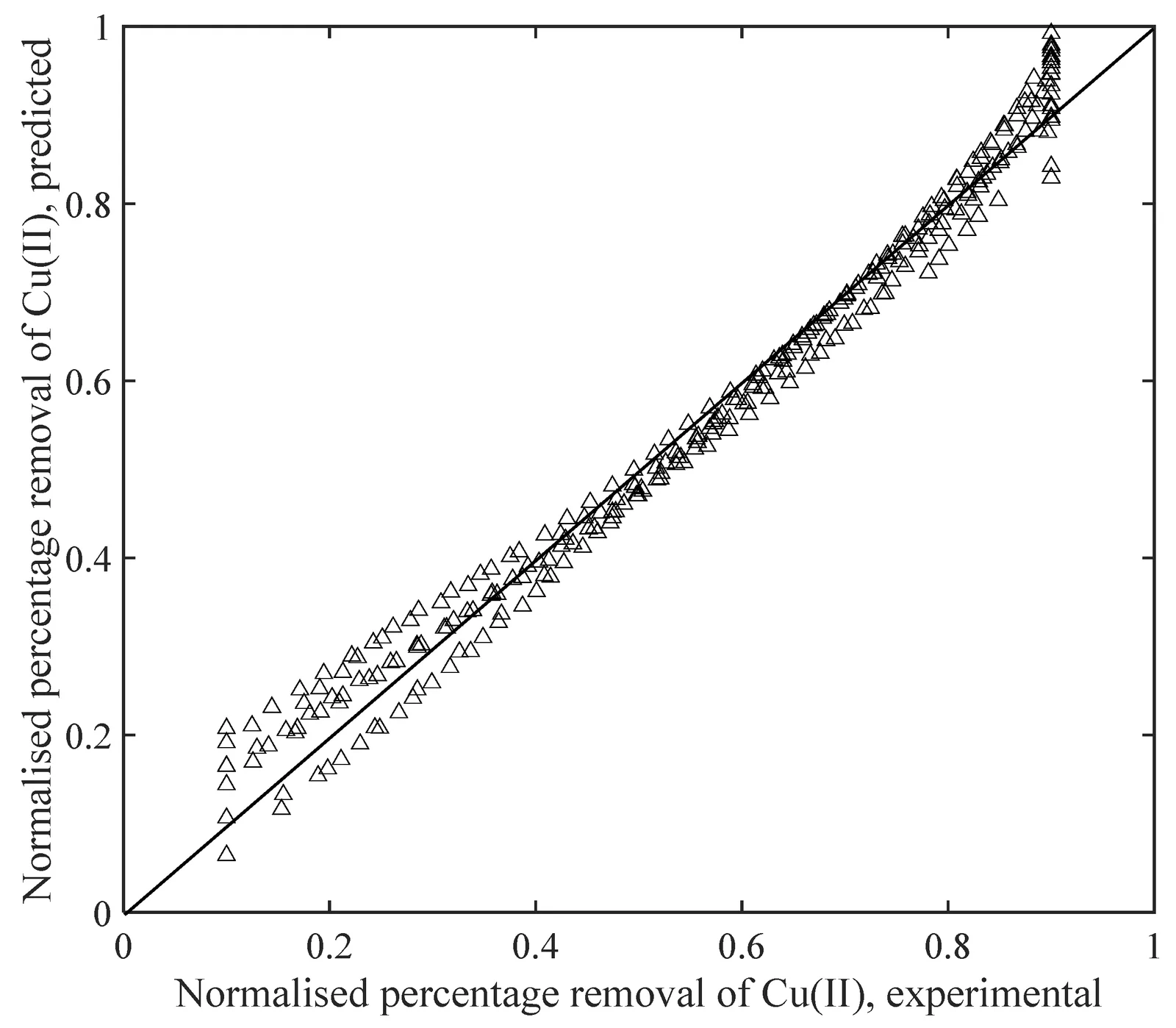
MLR comparison plot.

Figure 6 depicts the visualization of the comparison of normalized percentage removals associated with the experimental and predicted data, respectively.

### 3.8. Data Analysis with a GA

In recent years, the application of GAs has led to the prediction of the percentage of removal of heavy metals from aqueous solutions [26,48].

The experimental data (used for the MLR) was replicated thrice with consecutive columns and rows to construct three samples, namely, R-Cu(II)_1_, R-Cu(II)_2_, and R-Cu(II)_3_. The input parameters are the same as presented in Table 3. Then, three sets were randomized independently of each other to minimize random errors that may occur during training. This step is also necessary for generalization. Then, the data necessary for the final prediction was segregated and maintained separate for all three samples [33].

Similarly, cross-validation data was segregated and maintained separate for all three samples. Lastly, 20% of the overall data for each set was maintained for training, while the training and cross-validation data was processed simultaneously. However, the process is limited to a maximum of 1000 epochs, and 100 iterations were permitted to improve the mean squared error related to cross-validation. If no improvement was observed, the training was halted. The network is considered optimized at the iteration when the lowest cross-validation error is obtained during the training with the corresponding weight values.

**Figure 7:**
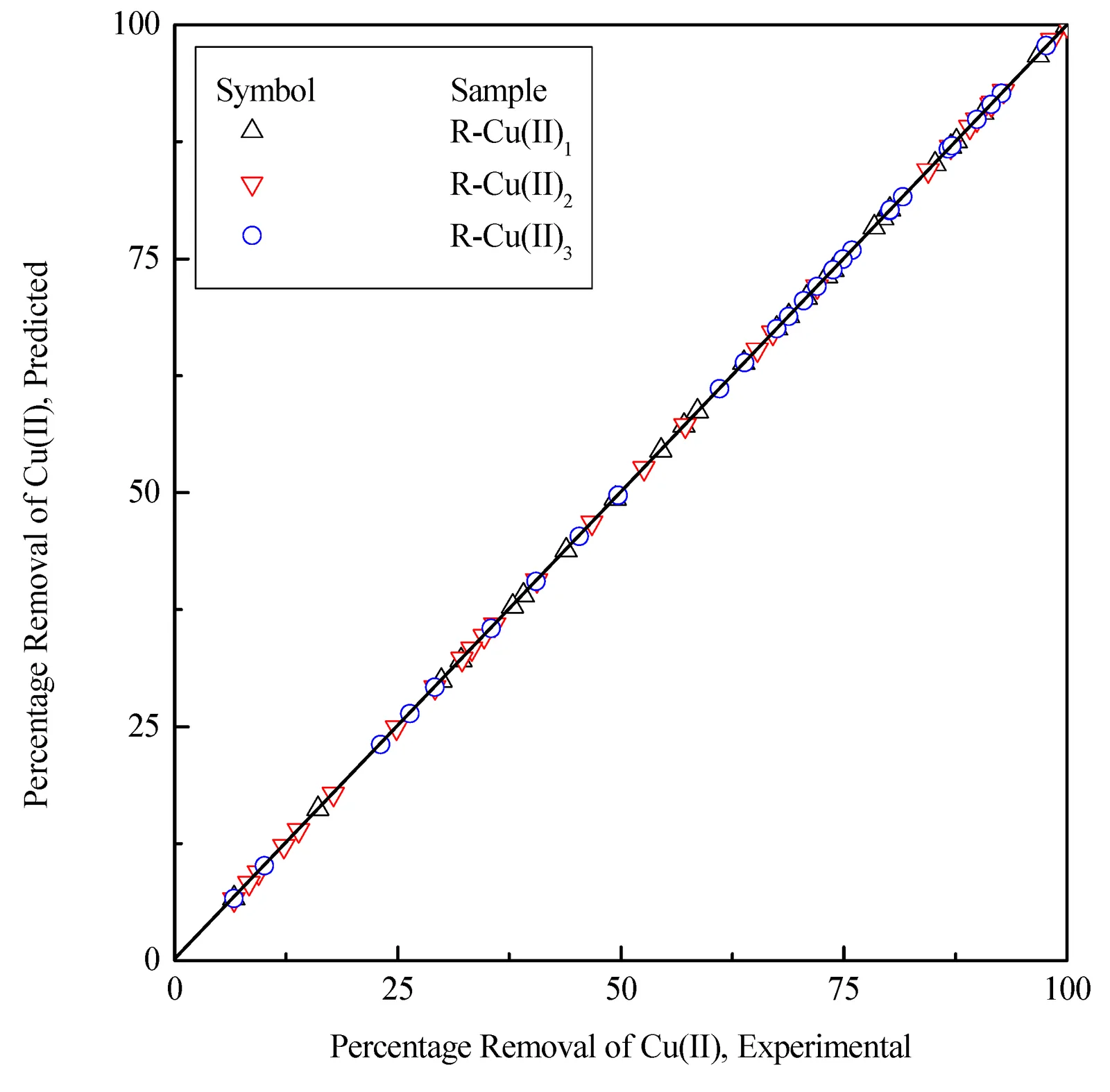
GA comparison plot.

The input parameters were optimized using the widely recognized Levenberg–Marquardt (LM) training algorithm. Subsequently, a Genetic Algorithm (GA) was applied to identify the generation associated with the minimum generation error. During the optimization process, the chromosomes were updated using a mutation probability of 0.1 and a crossover probability of 0.9 [18,25,26,33]. The optimization was considered to have converged when the generation error approached 10^−6^. The corresponding generation errors and optimization results obtained from the GA analysis are presented in Table 4. The results in Table 4 and Figure 7 indicate that an R-value was achieved for the investigated samples, demonstrating excellent agreement between the predicted and experimental values. These findings support the effectiveness of the model in predicting the percentage removal.

The final results obtained using the multiple linear regression (MLR) and Genetic Algorithm (GA) techniques demonstrate that both approaches can reliably predict the percentage removal. However, a comparison of their respective correlation coefficients reveals that the GA-based model exhibits a slightly higher predictive accuracy than the MLR model. This suggests that the GA provides a marginally better performance in predicting percentage removal under the investigated experimental conditions.

### 3.9. Comparability Study

The adsorption capacities for Cu(II) based on the Thomas model of different adsorbents reported in previous studies are tabularized in Table 5. The comparison table establishes the usefulness of the adsorbent employed in the current work.

## 4. Conclusions

Chitosan–nanosilica (*n*SiO_2_) nanocomposites were synthesized and used to remove the Cu(II) ions from an aqueous solution. Here, *n*SiO_2_ was synthesized from rice husk ash. The effects of various operating parameters in fixed-bed studies were investigated. The outcomes can be summarized as follows:

1. The optimum pH of 6.5, calculated during the batch study, was employed for the column study. The removal of Cu(II) from aqueous media under continuous-flow conditions was investigated by examining the effects of adsorbent bed depth, influent flow rate, and influent Cu(II) concentration.
2. The Cu(II) removal efficiency increased with increasing bed depth, whereas it decreased with increasing influent flow rate and initial Cu(II) concentration.
3. The Thomas model provided a satisfactory description of the adsorption kinetics, demonstrating a good correlation between the experimental and predicted data.
4. The scale-up design of the adsorption column demonstrated the potential of CSNC2-1 as a cost-effective adsorbent for the large-scale treatment of Cu(II)-contaminated wastewater in industrial applications.
5. Genetic Algorithm (GA) and multiple linear regression (MLR) techniques were successfully applied to the experimental data to model and predict the adsorption performance.

Overall, the performance of the prepared adsorbent was evaluated on a laboratory scale for removing Cu(II) from synthetic wastewater, demonstrating its potential for application in continuous fixed-bed adsorption systems.

## Figures and Tables

**Table 1:** Kinetic models of column study.

Initial Cu(II) concentration (*C*_0_) mg L^−1^	10	10	10	20	30	10	10
Influent flow rate (*Q*) mL min^−1^	50	50	50	30	30	30	40
Bed height (*Z*) cm	3	5	7	3	3	3	3
Experimental Adsorption capacity, *q_e_*_(exp)_ (mg g^−1^)	39.786	32.421	28.809	53.692	50.567	42.224	39.874
Bohart–Adams model [42]							
Mathematical equation				Comments			
$\ln\left(\frac{C_{t}}{C_{0}}\right)=K_{\mathrm{BA}}C_{0}t-K_{\mathrm{BA}}N_{0_{\mathrm{BA}}}\left(\frac{Z}{U_{0}}\right)$				This mathematical model is used to describe the initial stage of the adsorption process. It explains the relationship between the adsorption capacity of the adsorbent, the *C*_0_, and the breakthrough behavior. It assumes that the adsorption rate is proportional to the residual adsorption capacity of the adsorbent and the concentration of the adsorbate in the liquid phase. *K_BA_* decreased with *C*_0_. $N_{0_{\mathrm{BA}}}$ decreased with *Z* but increased with *C*_0_ and *Q* [33].			
*K_BA_* (L mg^−1^ min^−1^)	0.000492	0.000517	0.000449	0.000348	0.000296	0.000514	0.000528
$N_{0_{\mathrm{BA}}}$ (g L^−1^)	42.6065	31.3785	25.8181	51.526	52.0659	39.4043	40.1832
R^2^	0.92869	0.77892	0.90747	0.9192	0.91374	0.80213	0.84427
SSE	1.36708	9.81279	3.28426	2.86535	1.70044	15.25215	5.2897
SD	0.20669	0.50161	0.27964	0.30402	0.28456	0.5637	0.38332
Thomas model [43]							
Mathematical equation				Comments			
$\ln\left(\frac{C_{0}}{C_{t}}-1\right)=\frac{K_{\mathrm{Th}}q_{\mathrm{Th}}m}{Q}-K_{\mathrm{Th}}C_{0}t$				This model is based on Langmuir adsorption–desorption kinetics, in which the rate expression follows reversible reaction kinetics. It assumes that axial dispersion within the column is negligible. *K_Th_* decreased with increasing *Z* but increased with increasing *Q*, whereas *q_Th_* decreased with Z and *Q* [33].			
Second-order reaction kinetics. *K_Th_* (mL mg^−1^ min^−1^)	1.009	0.921	0.79	0.595	0.5313	0.805	0.936
*q_Th_* (mg g^−1^)	39.48	32.68	29.14	53.85	51.18	43.74	40.49
R^2^	0.97355	0.9187	0.96714	0.96111	0.98	0.93725	0.95348
SSE	1.86246	9.00401	3.16234	3.51905	1.0335	9.5574	4.05934
SD	0.24511	0.48677	0.27772	0.34249	0.22732	0.45094	0.34056
Wolborska model [44]							
Mathematical equation				Comments			
$\ln\left(\frac{C_{t}}{C_{0}}\right)=\frac{\beta_{W}C_{0}t}{N_{0_{W}}}-\frac{\beta_{W}Z}{U_{0}}$				This is an empirical model used to describe the initial stage of a breakthrough curve and is particularly useful for analyzing the mass transfer process and estimating the external mass transfer coefficient during the early stages of adsorption. *β_W_* increased with an increase in *Q* and decreased with an increase in *C*_0_ and *Z.* $N_{0_{W}}$ increased with *C*_0_ and *Q* rate but decreased with *Z* [33].			
*β_W_* (min^−1^)	21.6584	16.2227	11.5923	17.931	15.4289	20.2538	21.2167
$N_{0_{W}}$ (g L^−1^)	44.0212	31.3785	25.8181	51.526	52.0658	39.4043	40.1831
R^2^	0.92869	0.77892	0.90747	0.9192	0.91374	0.80213	0.84427
SSE	1.36708	9.81279	3.28426	2.86535	1.70044	15.25215	5.2897
SD	0.20669	0.50161	0.27964	0.30402	0.28456	0.5637	0.38332
Yoon–Nelson model [45]							
Mathematical equation				Comments			
$\ln\left[\frac{C_{t}}{\left(C_{0}-C_{t}\right)}\right]=k_{\mathrm{YN}}t-\tau_{\mathrm{YN}}k_{\mathrm{YN}}$				This is a widely used empirical model for describing the breakthrough behavior. It assumes that the rate of decrease in the probability of adsorption is proportional to the probability of adsorbate breakthrough on the adsorbent surface. *τ_YN_* decreased with increasing *Q* and *C*_0_ but increased with *Z*. *K_YN_* increased with *C*_0_ and *Q* but decreased with increasing *Z* [33].			
*K_YN_* (min^−1^)	0.01009	0.00921	0.0079	0.0119	0.01594	0.00805	0.00936
*τ_YN_* (min)	277.6293	368.0945	433.0886	315.6227	199.9686	512.7193	355.9274
R^2^	0.97355	0.9187	0.96714	0.96111	0.98	0.93725	0.95348
SSE	1.86246	9.00401	3.16234	3.51905	1.0335	9.5574	4.05934
SD	0.24511	0.48677	0.27772	0.34249	0.22732	0.45094	0.34056
Yan et al.’s model [46]							
Mathematical equation				Comments			
$\ln\left[\frac{C_{t}}{\left(C_{0}-C_{t}\right)}\right]=\frac{K_{Y}C_{0}}{Q}\ln\left(\frac{Q^{2}}{K_{Y}q_{Y}m}\right)+\frac{K_{Y}C_{0}}{Q}\ln t$				This is an empirical model developed to overcome certain limitations of the Thomas model, particularly its difficulty in accurately predicting the effluent concentration near the initial stage of the adsorption process. It provides a flexible representation of breakthrough curves and can be used to evaluate the influence of operating parameters on column performance. *K_Y_* increased with increasing *Z* and *Q* but decreased with increasing *C*_0_. *q_Y_* increased with increasing *Q* and *C*_0_ but decreased with increasing *Z* [33].			
*K_Y_* (mL mg^−1^ min^−1^)	9.273	10.33415	11.10475	3.25218	1.78644	8.45142	8.8144
*q_Y_* (mg g^−1^)	0.3348	0.1351	0.0778	0.2748	0.8257	0.0654	0.1652
R^2^	0.85279	0.87604	0.90368	0.86898	0.87686	0.98254	0.92387
SSE	10.36571	13.72757	9.27024	11.85534	6.3619	2.65975	6.6439
SD	0.57825	0.60104	0.4755	0.62863	0.564	0.23789	0.43569
Modified dose–response model [46]							
Mathematical equation				Comments			
$\ln\left[\frac{C_{t}}{\left(C_{0}-C_{t}\right)}\right]=a_{\mathrm{mdr}}\ln\left(C_{0}\mathrm{Qt}\right)-a\ln\left(q_{\mathrm{mdr}}m\right)$				This empirical model relates the effluent concentration ratio (*C*_t_/*C*_0_) to the operating time and column parameters. The model is useful for analyzing experimental breakthrough curves and predicting the performance of continuous adsorption systems under different operating conditions, including variations in *Z*, *Q*, and *C*_0_. The model parameters are estimated by fitting the equation to the experimental data. *q_mdr_* decreased with increasing *Q* and *Z*. *a_mdr_* decreased with *C*_0_ and *Q* but increased with *Z* [33].			
*a_mdr_*	1.8546	2.0668	2.221	2.1681	1.7864	2.8171	2.2036
*q_mdr_* (mg g^−1^)	32,559.02	28,228.4	26,188.47	48,858.24	44,409.89	39,509.18	35,547.69
R^2^	0.85279	0.87604	0.90368	0.86898	0.87686	0.98254	0.92387
SSE	10.36571	13.72757	9.27024	11.85534	6.3619	2.65975	6.6439
SD	0.57825	0.60104	0.4755	0.62863	0.564	0.23789	0.43569
Bed depth service time model [47]							
Mathematical equation				Comments			
$t=\frac{N_{0_{\mathrm{bdst}}}Z}{C_{0}U_{0}}-\frac{1}{K_{\mathrm{bdst}}C_{0}}\ln\left(\frac{C_{0}}{C_{t}}-1\right)$				This is an empirical model used to describe and predict the performance of fixed-bed adsorption column systems. It establishes the relationship between the *Z* of an adsorbent and the service time required to reach a specific breakthrough concentration of the adsorbate in the effluent. It provides a preliminary basis for designing large adsorption columns using laboratory-scale experimental data, provided operating conditions and model assumptions remain applicable. The values of $N_{0_{\mathrm{bdst}}}$ and *K_bdst_* increased with increasing $\frac{C_{t}}{C_{0}}$ values [33].			
$\frac{C_{t}}{C_{0}}$	$N_{0_{\mathrm{bdst}}}$ (g L^−1^)		*K_bdst_* (L mg^−1^ min^−1^)	R^2^		SSE	SD
0.1	5.26964		−0.204392984	0.98868		31.74	5.7318
0.5	11.8832		0	0.99648		49.82402	7.0586
0.9	12.5117		0.000609704	0.99818		28.47082	5.3358

**Table 2:** Scale-up design for adsorption of Cu(II) by CSNC2-1.

W (m^3^)	*t_W_* (min)	C_w_ (mg L^−1^)	Q_d_ (L min^−1^)	D (m)	M_ad_ (kg)	H_c_ (m)	H (m)
10	360	30	27.78	0.456	0.236	0.25	0.252
20	600	30	33.33	0.5	0.283	0.35	0.352

**Table 3:** Description of data for MLR.

Parameter	Range
Column diameter, *D_c_* (m)	0.015
Flow rate, *Q* (mL min^−1^)	30–50
Bed height, *Z* (m)	0.03–0.07
Time, *t* (min)	0–810
Influent Conc., *C_O_* (mg L^−1^)	10–30
Final Conc., *C_t_* (mg L^−1^)	0–30
Percentage removal, $PR_{\mathrm{Col}-{\mathrm{SiO}}_{2}-\mathrm{Cu}(\mathrm{II})}$ (%)	0–100

**Table 4:** Optimization of GA network performance.

Randomization No.	Minimum Generation Error	AARE	SD (σ)	MSE	CCC (R)
R-Cu(II)_1_	4.0447 × 10^−8^	0.00029	0.0004	0.00024	1
R-Cu(II)_2_	1.0103 × 10^−7^	0.00028	0.00025	0.00038	1
R-Cu(II)_3_	3.9035 × 10^−7^	0.00169	0.00389	0.00132	1

**Table 5:** Comparison of column adsorption capacities based on the Thomas model.

Adsorbents	Adsorption Capacities for Cu(II) in mg g^−1^	Reference
Yersiniabactin, immobilized to XAD16 resin	0.12	[49]
Coal fly ash	11.39	[50]
Silicate tailings	11.65	[51]
10% pruned bamboo BBm	0.08	[52]
Almond shell	3.90	[41]
Chitosan-coated bentonite	12.14	[53]
Chitosan immobilized on bentonite	40.38	[54]
Chitosan-based fibrous materials	11.16	[55]
Modified chitosan hydrogel	48.0	[56]
CSNC2-1	53.85	Present study

## Data Availability

Data are available upon reasonable request.

## Abbreviations

*a_mdr_*	Modified dose–response kinetic model parameter
C_0_	Influent Cu(II) concentration (mg L^−1^)
C_d_	Cu(II) discharge limit for inland surface water (mg L^−1^)
C_t_	Effluent Cu(II) concentration at time t (mg L^−1^)
C_w_	Cu(II) concentration in wastewater (mg L^−1^)
D	Diameter of the adsorption column (m)
H	Total bed height in the column (m)
H_c_	Additional height for accessories (m)
*K_BA_*	Kinetic constant in Bohart–Adams model (L mg^−1^ min^−1^)
*K_bdst_*	Kinetic constant in bed depth service time model (L mg^−1^ min^−1^)
*K_YN_*	Kinetic constant in Yoon–Nelson kinetic model (L mg^−1^ min^−1^)
*K_Th_*	Kinetic constant in Thomas model (mL mg^−1^ min^−1^)
*K_Y_*	Kinetic constant in Yan et al.’s model (mL mg^−1^ min^−1^)
M_ad_	Daily designed adsorbent quantity (kg)
*m_d_*	Mass of dry adsorbent in column (g)
m_total_	Mass of the adsorbate added to the column (mg)
$N_{0_{\mathrm{BA}}}$	Bohart–Adams model adsorption capacity of bed (mg L^−1^)
$N_{0_{W}}$	Wolborska model adsorption capacity of bed (mg L^−1^)
$N_{0_{\mathrm{bdst}}}$	Bed depth service time model adsorption capacity (g L^−1^)
Q	Volumetric flow rate (L min^−1^)
Q_d_	Designed volumetric flow rate (L min^−1^)
*q_ad_*	Adsorptive capacity of the adsorbent as per the best-fit model (mg g^−1^)
*q_e_*_(exp)_	Experimental adsorption capacity (mg g^−1^)
*q_mdr_*	Modified dose–response model maximum adsorption capacity (mg g^−1^)
*q_R_*	Amount of Cu(II) adsorbed after regeneration (mg)
*q_Th_*	Thomas model maximum adsorption capacity (mg g^−1^)
*q_Y_*	Yan et al.’s model maximum adsorption capacity (mg g^−1^)
*q_total_*	Total Cu(II) adsorbed (mg)
t	Time (min)
t_R_	Bed regeneration time (min)
t_total_	Total flow time (min)
*t_W_*	Working time (min)
U_0_	Superficial velocity (m min^−1^)
V_eff_	Effluent volume (L)
W	Daily wastewater flow (m^3^)
Z	Bed depth (cm)
Greek letters	
*β_W_*	Kinetic coefficient of external mass transfer in Wolborska model (min^−1^)
*ε*	Regeneration efficiency
*ρ_ad_*	Density of the adsorbent (kg m^−3^)
*τ_YN_*	Half-life of adsorbate (min)
